# The Challenges of Screening Mammography in Racial/Ethnic Minority Populations in the United States: A mini-review and observations from a predominantly Hispanic community

**Published:** 2018-04-05

**Authors:** Julia E. McGuinness, Katherine D. Crew

**Affiliations:** 1 Department of Medicine, College of Physicians and Surgeons, Columbia University, New York, NY 10032; 2 Department of Epidemiology, Mailman School of Public Health, Columbia University, New York, NY 10032; 3 Herbert Irving Comprehensive Cancer Center, Columbia University, New York, NY 10032

**Keywords:** Breast cancer, Screening mammography, False positive results, Hispanic, Epidemiology

## Abstract

Screening mammography is recommended by U.S. medical organizations for breast cancer screening in average risk women because of its demonstrated reductions in breast cancer mortality. However, significant disparities in breast cancer screening utilization and mortality remain among racial/ethnic minorities. Efforts have appropriately been directed at increasing engagement with screening services in these populations, however, there is a dearth of data regarding false-positive rates and overdiagnosis in minority patients engaged in breast cancer screening. We recently examined screening practices among a predominantly Hispanic population presenting to an academic medical center in New York, NY, and found that approximately 53% of women experienced at least one false-positive mammography result over a median of 8.9 years of screening. We also observed that Hispanic women were more likely to screen annually than white women despite recommendations to screen less frequently. In this review, we briefly review the benefits and harms of screening mammography in average-risk women, namely, false-positive results and breast cancer overdiagnosis, followed by a discussion of the disparities in breast cancer screening and mortality among racial/ethnic minority populations. We then present our own recent observations and propose that future interventions among Hispanic and other minority populations could include patient- and provider-centered educational programs that focus on providing a balanced discussion of benefits and harms of screening mammography.

## Screening Mammography in Average-Risk Women: A Brief Summary of Benefits and Harms

Screening mammography has been recommended as a screening tool for breast cancer for decades, and since the initiation of population-based mammographic screening in the 1980s in the United States, breast cancer mortality has decreased steadily by 30 to 40 percent, partly due to early detection with screening^1^. Multiple European and American studies have demonstrated mortality benefits to screening mammography, and as a result, most U.S. major professional organizations recommend its use for breast cancer screening^2–8^. Advancements in screening technology, including the development of digital mammography to replace screen-film mammography and digital breast tomosynthesis (or “three-dimensional mammography”), are expected to further contribute to reductions in breast cancer mortality.

While the benefits of screening mammography are almost universally accepted by healthcare professionals, in recent years there has been an increasing focus on the potential physical, psychological, and financial harms of mammography. This interest is largely centered on the rates of false positive mammography results and breast cancer overdiagnosis. False positive mammography is defined as recall breast imaging and/or breast biopsy that does not yield a diagnosis of breast cancer, while overdiagnosis is defined as a screen-detected breast cancer that would not have led to symptomatic cancer if it had not been detected on imaging, (*i.e.,* ductal carcinoma *in situ* (DCIS) or small and early-stage breast cancers). The cumulative probabilities of false positive findings from screening mammography over 10 years of annual screening in the United States is as high as 61%, and can be up to 16% for a woman’s first mammogram^2,9–12^. The data range widely for estimated rates of breast cancer overdiagnosis, from 5% to 50%; contributing to this variability is the lack of consensus on the definition of overdiagnosis and how to measure it^13^. In addition to physical harms from unnecessary testing, procedures, or treatment, women can also experience significant psychological distress with false positive results. Some studies showed similar levels of distress in women with false positive results after screening mammography to those who received a diagnosis of breast cancer, and consistently higher scores of psychological distress in women with false positive results, persisting up to 12 months^14–17^. Personal and national financial costs can also be substantial. In the United States between 2012 and 2013, the cost of false-positive results from screening mammography was 2.8 billion dollars, with an average $200 out of pocket cost to patients^18^.

The particular challenge of reducing rates of overdiagnosis or false positive mammography results while still maintaining the mortality benefits of screening mammography has gained national attention. Investigation into factors affecting rates of false positives has revealed areas for possible improvement. Decreasing the screening frequency from annual to biennial mammography decreases the cumulative probability of a false positive result to 29 to 42% without significantly affecting mortality reduction^9,12^. Younger age (particularly age 40–45), dense breasts, and breast cancer risk factors have also been found to increase the probability of a false positive result^19^. Taking into account this data, major U.S. medical organizations have modified their screening recommendations regarding the ages of onset as well as frequency of mammography screening for women at average risk of breast cancer, without a clear consensus among them^3,4,6–8^. Recommendations range from biennial screening for all starting at age 50, to annual screening starting at age 40.

## Disparities in Breast Cancer Screening among Racial/Ethnic Minority Populations

Discussions regarding how to minimize rates of false-positive mammography and breast cancer overdiagnosis have applied predominantly to non-minority populations with adequate access to healthcare. In contrast, efforts in minority and underserved populations have been focused on increasing access to and utilization of screening mammography programs. Racial/ethnic minority populations in the United States, including blacks, Hispanics, and American Indians, consistently have lower utilization of screening mammography when compared to non-Hispanic white populations^20–24^. Geographic variations in utilization of screening mammography by minority populations exist; for example, among Medicaid-insured women in 44 states, black women were less likely to use screening mammography resources than white women in 13 states, but more likely than white women to use mammography in six states^25^. Minority women are also more likely to be diagnosed with breast cancer at more advanced stages, possibly because of inadequate use of screening mammography, and breast cancer mortality is higher among minorities, particularly among blacks^26–30^. However, providing more equal access to healthcare and screening resources has the potential to improve these disparities. In the Department of Defense Healthcare System, an equal-access model of healthcare with standard screening practices, the racial/ethnic differences in utilization of screening mammography are not observed, and survival from early stage breast cancer is not different between black and white women^31,32^.

Numerous local and national programs have been implemented to address these racial/ethnic disparities in breast cancer. An example of successful national efforts to improve access to cancer screening in minority women is the National Breast and Cervical Cancer Early Detection Program (NBCCEDP). In 1990, the U.S. Congress enacted the Breast and Cervical Cancer Mortality Prevention Act, which authorized the Center for Disease Control (CDC) to establish the NBCCEDP, a nationwide, comprehensive public health program with the mission of increasing access to breast and cervical cancer screening for women who were medically underserved^33^. The estimated life-years saved by NBCCEDP between 1991 and 2006 was 100,800 compared with no program and 369,000 life-years compared with no screening^34^. One of the objectives of the program was to target the racial/ethnic disparities in screening, diagnosis, and treatment of breast and cervical cancers. Of the 978,382 women screened through NBCCEDP between July 2011 and 2016 across the U.S., approximately 60% were from racial/ethnic minority groups^35^. The strategies employed to increase screening and breast cancer treatment among racial/ethnic minorities were diverse, and include reminders for patients, culturally-tailored programs that address specific beliefs or knowledge gaps, and programs addressing financial or logistical barriers to screening^36^. On a more local level, the Metropolitan Chicago Breast Cancer Taskforce was formed in 2007 in response to progressive disparities in breast cancer mortality between black and white populations in Chicago, which had resulted in a black breast cancer mortality rate 116% higher than the white rate^37^. Hypothesizing that black women received fewer mammograms, mammograms of inferior quality, and lower quality treatment for breast cancer once diagnosed, various public health and public policy initiatives were implemented. Between 2006 and 2013, the disparity in mortality rates between non-Hispanic blacks and non-Hispanic whites decreased by 20%, even as this disparity increased in other U.S. cities^38^.

Increasing access to screening mammography is a vital part of improving breast cancer morbidity and mortality in minority populations, but minimizing the potential harms of screening in this population is, as in the general population, a challenge. While there is a dearth of studies specifically exploring false positive mammography results and overdiagnosis in minority populations, in an analysis of screening data at facilities serving “vulnerable” populations with overall lower educational attainment, a greater proportion of racial/ethnic minorities, and lower income, rates of false positive mammograms were significantly higher when compared with rates at facilities not serving vulnerable populations, without clear explanation^39^. The psychological harms of false positive results are of particular concern in this population because of their potential to decrease adherence to continued screening. After receiving false-positive results on screening mammography, only 71% of Hispanic women and 80% of black women were likely or very likely to continue screening mammography, compared with 93% of white women. Only 59% of Hispanic women and 53% of black women were willing to return for recall imaging even if it meant a higher cancer detection rate, compared with 76% of white women^40^.

## Our Observations in a Predominantly Hispanic Population, and Proposed Future Interventions

There is some evidence that Hispanic women in particular have a tendency to more frequently utilize screening mammography than other racial/ethnic minority groups when they have access to screening resources. In an analysis of the screening patterns of approximately 2.5 million female Medicaid beneficiaries in 25 states, Hispanic women, unlike American Indian or black women, had higher odds ratio of mammography use than white women^41^. This is despite Hispanic women as a group having lower incidences of invasive breast cancer than non-Hispanic white and blacks^42^. The concern with more frequent annual screening in this population is that it will lead to further increases in false positive rates, and potentially to premature discontinuation of appropriate screening practices in Hispanic women who have received false positive results. As such, there is a need to more closely examine screening patterns in this population in order to maximize benefits of mammography while mitigating harms.

In the Washington Heights neighborhood of northern Manhattan surrounding Columbia University Medical Center (CUMC), the population is predominantly Hispanic, with Caribbean origins. We sought to investigate the rates of false positive mammography results in this predominantly minority, Hispanic population, as well as factors associated with higher rates of false positive results, for the reasons previously detailed. We found that among the largely Hispanic (70%) population of women presenting for screening mammography at the Avon Breast Imaging Center at CUMC and agreeing to participate in our study, approximately 53% of women had at least one false positive result on their screening mammograms over a median of 8.9 years of screening (range, 0–26)^43^. The factors associated with false positive results were similar to those of more predominantly white, non-minority populations, namely, frequency of screening and increased breast density. Women who screened annually compared to biennially were over two times more likely to have a false positive result (odds ratio [OR]=2.18; 95% confidence interval [CI]=1.70–2.80). Moreover, 69% of women at the center were still screening annually despite the changes in national guidelines in the past decade favoring less frequent screening in average risk women. Hispanic women were more likely to undergo annual screening mammography than non-Hispanic white women (OR=1.92; 95% CI= 1.17–3.15). This finding is consistent with the previously-mentioned data among minority Medicare recipients, and while we recruited from a patient population already actively engaged in medical care at an academic institution, it underscores a potential need to address the apparent overutilization of screening mammography.

Providers serving Hispanic women might therefore face the challenge of engaging them in breast cancer screening and encouraging their compliance, but also avoiding potential overutilization in those actively utilizing screening resources. One potential approach is to identify culturally-specific knowledge gaps or beliefs and to develop patient- and provider-centered decision support tools. Our research group is currently developing and investigating a web-based patient-centered decision aid and provider-centered decision support tool to help educate patients and providers not only on the importance of breast cancer screening and the current guidelines, but also on breast cancer risk assessment and minimizing harms including false-positive results and overdiagnosis. Educational interventions designed to increase screening rates among Hispanic women have shown success, with demonstrated increases in screening in several randomized control trials, particularly when interventions were multicomponent and involved both community health workers and clinic-level interventions^44^. Future studies could assess the effectiveness of educational interventions providing information about personal breast cancer risk and recommended screening practices in improving long-term compliance with screening and even perhaps in reducing false-positive results. We use the Hispanic population as an example because of our own observations and proposed interventions in a predominantly-Hispanic population presenting for screening mammography. These lessons could be applied to other racial/ethnic minorities, with careful attention to their unique cultural identities and needs as well as their screening practices.

In conclusion, disparities in screening mammography utilization continue to exist among racial/ethnic minority groups, and efforts must continue to be directed toward increasing access to and compliance with screening in these populations. However, given the potential harms of screening mammography (*i.e.,* false-positive results and breast cancer overdiagnosis), once racial/ethnic minority women are established in screening programs efforts should also be directed toward encouraging a risk-stratified approach to screening.
